# Chromosome-level genome assembly and annotation of the yellow grouper, *Epinephelus awoara*

**DOI:** 10.1038/s41597-024-02989-8

**Published:** 2024-01-31

**Authors:** Weiwei Zhang, Yang Yang, Sijie Hua, Qingxin Ruan, Duo Li, Le Wang, Xi Wang, Xin Wen, Xiaochun Liu, Zining Meng

**Affiliations:** 1 State Key Laboratory of Biocontrol, Institute of Aquatic Economic Animals and Guangdong Province Key Laboratory of Aquatic Economic Animals, School of Life Sciences, Sun Yat-sen University, Guangzhou, 510275 China; 2 Key Laboratory of Tropical Marine Fish Germplasm Innovation and Utilization, Ministry of Agriculture and Rural Affairs, Sanya, 570000 China; 3 Hainan Engineering Research Center for Germplasm Innovation and Utilization, Sanya, 570000 China; 4 Molecular Population Genetics Group, Temasek Life Sciences Laboratory, National University of Singapore, Singapore City, 119077 Singapore; 5 Area of Ecology and Biodiversity, School of Biological Sciences, University of Hong Kong, Hong Kong SAR, 999077 China; 6 School of Marine Biology and Fisheries, Hainan Aquaculture Breeding Engineering Research Center, Hainan Academician Team Innovation Center, Hainan University, Haikou, 570228 China; 7 Southern Laboratory of Ocean Science and Engineering (Zhuhai), Zhuhai, 519000 China

**Keywords:** Comparative genomics, Evolutionary genetics

## Abstract

*Epinephelus awoara*, as known as yellow grouper, is a significant economic marine fish that has been bred artificially in China. However, the genetic structure and evolutionary history of yellow grouper remains largely unknown. Here, this work presents the high-quality chromosome-level genome assembly of yellow grouper using PacBio single molecule sequencing technique (SMRT) and High-through chromosome conformation capture (Hi-C) technologies. The 984.48 Mb chromosome-level genome of yellow grouper was assembled, with a contig N50 length of 39.77 Mb and scaffold N50 length of 41.39 Mb. Approximately 99.76% of assembled sequences were anchored into 24 pseudo-chromosomes with the assistance of Hi-C reads. Furthermore, approximately 41.17% of the genome was composed of repetitive elements. In total, 24,541 protein-coding genes were predicted, of which 22,509 (91.72%) genes were functionally annotated. The highly accurate, chromosome-level reference genome assembly and annotation are crucial to the understanding of population genetic structure, adaptive evolution and speciation of the yellow grouper.

## Background & Summary

Groupers belongs to the subfamily Epinephelinae under the family Epinephelidae, which mainly inhabits tropical and subtropical coral reefs or continental shelves, acting as the top predators maintaining the ecological balance of coral reef ecosystems^1^. Groupers encompasses over 16 genera and more than 160 species, out of which approximately 47 species were currently cultivated for aquaculture^2^, making them globally significant economic fish species. According to statistics from the Food and Agriculture Organization (FAO), the global aquaculture production of groupers in 2020 amounted to 226.2 thousand tonnes^3^. In China, as per data from the China Fisheries Statistical Yearbook 2023, the aquaculture production of groupers in 2022 reached 205.8 thousand tonnes, ranking it fourth in terms of marine aquaculture production in China^4^. In conclusion, groupers hold significant ecological aimportance and commercial value. In recent years, several high-quality chromosome-level assemblies of grouper reference genomes have been assembled, including seven species of the *Epinephelus* genus^5–13^, one species of the *Plectropomus* genus^14–17^, one species of the *Cromileptes* genus^18^, and one species of the *Cephalopholis* genus^19^, which provide important genomic resources for evolutionary analysis, molecular-assisted breeding, and germplasm conservation of groupers. Phylogenetic studies show that *Epinephelus* species in the western Pacific Ocean have diverged into two genetic clades with variations in body size^20,21^, implying the potential presence of independent evolutionary trajectories during the course of adaptation. However, a significant limitation exists, as reference genomes are available for seven species within the large-bodied clade^5,7–13,18^, whereas the small-bodied clade has an accessible reference genome for only one specie, the *E. akaara*^6^. This limitation hampers research on the body size differentiation, speciation, and evolutionary history of the groupers.

The subject of this study, *E. awoara*, commonly referred to as the yellow grouper, is widely distributed in occurs in the western North Pacific Ocean. Its distribution spans regions such as North and South Korea, Japan, China, Vietnam, and islands in the South China Sea, with occasional occurrences in the eastern Indian Ocean^22^. Notably, yellow grouper is one of the few grouper species capable of inhabiting higher latitudes, approximately around the 40th parallel north (40 °N). It is worth noting that the yellow grouper is a protogynous hermaphroditic fish^23^, belongs to the small-sized clade of *Epinephelus* species in the western Pacific Ocean^20,21^. It serves as valuable material for exploring sex change and body size differentiation in groupers. Distinguished by its striking appearance, characterized by a golden-yellow color on its abdomen, with five distinct banding patterns and prominently gilded outlines on the dorsal and caudal fins (Fig. 2b), yellow grouper has gained recognition and popularity among consumers and aquaculturists due to its delectable taste and considerable economic value. Presently, this species has been successfully subjected to artificial aquaculture in China, and has been utilized in hybrid breeding programs^24^. Consequently, it is considered as an economically promising fish species in the market. Despite broad research interests in this species, such as reproductive biology^23^, artificial breeding^24,25^, cytogenetics^26^, genetic structure^27,28^, and phylogenetics^29^, studies related to its genome-based breeding, evolution, and germplasm resources have been limited. Notably, only one report on the complete mitochondrial (mt) genome was available has delved into genetic resources^29^. Therefore, the assembly of a high-quality genome for yellow grouper is of paramount significance, and would provide an essential genome resource for molecular genetics, molecular breeding, evolutionary studies, and germplasm conservation of yellow grouper.

**Fig. 1:**
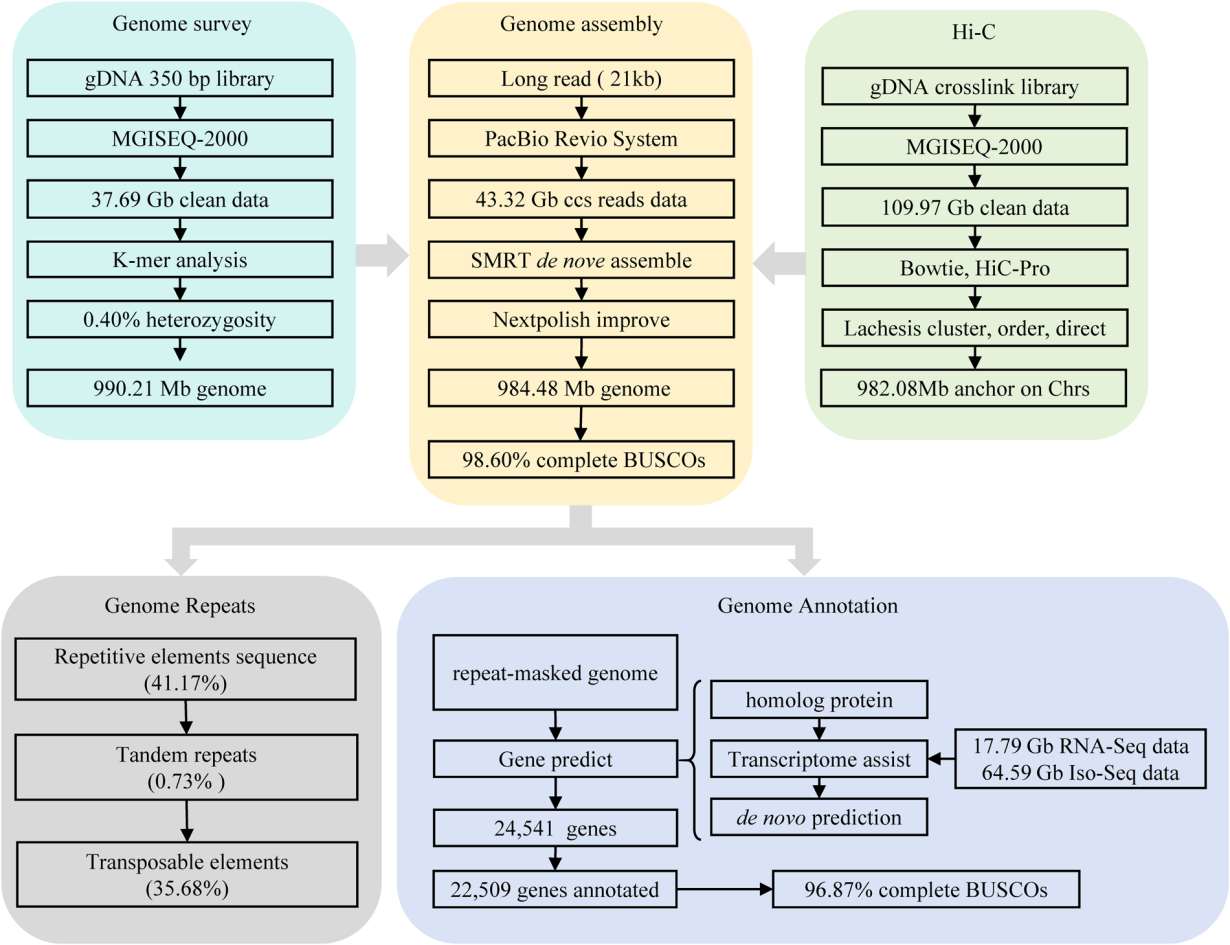
The overview of the chromosome-level genome assembly and annotation. Chrs: chromosomes. We first used 56.81 Gb short-read sequencing data to predict the assembled genome size was approximately 990.21 Mb Gb by K-mer analysis, and the GC content and heterozygosity were approximately 41.47% and 0.40%, respectively. Then, the 43.32 Gb of PacBio ccs data resulted in a 984.48 Mb assembly covers 98.60% of the genome, with contig N50 of 40.27 Mb. The 40 contigs were anchored into 24 pseudo-chromosomes covering roughly 99.76% of the genome assembly with the assistance of 114.71 Gb Hi-C reads. The final assembly consisted of 24 pseudo-chromosomes that yielded 984.48 Mb of yellow grouper genome, with a contig N50 of 39.77 Mb and scafold N50 of 43.39 Mb. The genome contained 35.96% repeat sequences and 8,235 noncoding RNAs. Additionally, 22,509 (91.72%) genes were functionally annotated from a total of 24,541 predicted protein-coding genes by combination of RNAseq and ISO-Seq annotation, genome sequence, and homolog protein.

The third-generation sequencing technology can produce genomes with high integrity through single-molecule real-time (SMRT) sequencing technology and high-throughput chromatin conformation capture (Hi-C) technology can achieve genome assembly at the chromosome level^14,30–32^. Recently, Pacific Biosciences released a new highly accurate long-read sequencer called the Revio System. In this study, we have employed a combination of PacBio Revio long-read circular consensus sequence (ccs) data and Hi-C technology to generate a high-quality, chromosome-level assembly of the yellow grouper genome. The workflow of *de novo* genome assembly and annotation is shown in the Fig. 1. The highly accurate, chromosome-level reference genome is essential to support basic genetic studies and will be contribute to genetic structure, evolutionary studies and germplasm conservation for yellow grouper.

## Methods

### Ethics statement

All experiments were performed according to the Guidelines for the Care and Use of Laboratory Animals in China. All experimental procedures and sample collection methods were approved by the Institutional Animal Care and Use Committee (IACUC) of the School of Life Sciences, Sun Yat-sen University under approval No. SYSU-IACUS-2022-B0129.

### Sample collection, library construction and sequencing

A wild female yellow grouper (body mass: 221.0 g) was collected at coastal of Daya Bay, Huizhou, Guangdong (22°43′1.28″ N; 114°32′14.54″ E). However, in fact, under natural conditions, yellow grouper are protogynous hermaphrodites 23. The muscle was collected for DNA extraction and was used for short-read sequencing, long-read SMRT sequencing, and Hi-C sequencing. 11 different tissues (muscle, gill, skin, liver, intestine, spleen, kidney, head-kidney, heart, eye, and brain) were collected for RNA extraction, then mixed into a total RNA for RNA Sequencing. All samples were immediately frozen in liquid nitrogen and then stored at −80 °C.

### Library construction and sequencing

All the DNA extraction, library construction and sequencing procedures were performed by the Nextomics Biosciences (Wuhan, China) according to the manufacturer’s protocols. Briefly, high molecular weight genomic DNA was extracted from muscle for muscle for MGI sequencing and PacBio SMRT sequencing. The DNA quality and purity of the extracted DNA was monitored on 0.75% agarose gels and NanoDrop One UV-Vis spectrophotometer (Thermo Fisher Scientific, USA). At last, DNA concentration of 316.0 ng/µL was measured by Qubit Fluorometer (Invitrogen, USA). High-quality DNA was used for library preparation and high-throughput sequencing.

For short-read sequencing, the genomic DNA (gDNA) was randomly fragmented and then the library was prepared following the manufacturer’s instructions. The qualified libraries were sequenced on MGISEQ-2000 platform. A total of 56.71 Gb raw reads were generated for genome survey. The Q20 rate and Q30 rate were 98.39% and 94.60%, respectively, with an average coverage depth of 57.27× (Table 1).

**Table 1:** Statistics of the DNA/RNA sequence data used for genome assembly.

Library type	Platform	Tissue	Raw data (Gb)	Clean data (Gb)	Average Read Length (bp)	N50 Read Length (bp)	Q20 rate (%)	Q30 rate (%)	Average coverage (×)
WGS short reads	MGISEQ-2000	Muscle	56.71	52.64	—	—	98.39	94.60	57.27
ccs long reads	PacBio Revio System	Muscle	43.32	—	21,905	23,217	—	—	43.75
Hi-C	MGISEQ-2000	Muscle	114.71	109.97	—	—	99%	99.9	111.06
ISO-Seq	PacBio Sequel II	Pooled RNA	64.59	—	2210	2617	—	—	65.23
RNA-seq	MGISEQ-2000	Pooled RNA	17.79	17.69	—	—	97.03	91.58	17.96

Note: Genome size estimated by genome survey (990.21 Mb) were used for sequencing coverage calculation.

For PacBio sequencing, the gDNA was used to construct SMRTbell libraries according to PacBio’s standard protocol (Pacific Biosciences, CA, USA) using 20 kb preparation solutions^33^. The SMRTbell library construction, inculding the DNA shearing, repair ends, and ligation of DNA fragments with hairpin adapters to create circular templates for SMRT sequencing. The library was sequenced using the PacBio Revio System in circular consensus sequence (CCS) mode following the manufacturer’s instructions. The quality control of the raw data was performed using ccs software (https://github.com/PacificBiosciences/ccs) with parameter of “-min-passes 1-min-rq 0.99-min-length 100”. As a result, 1 SMRT cell was sequenced, and a total of 43.32 Gb PacBio CCS reads (coverage depth as 43.75×) were generated for the subsequent genome assembly. The average read length and N50 length of the subreads sequences were 21,905 bp and 23,217 bp, respectively (Table 1).

For the Hi-C sequencing, the fresh muscle sample was fixed with 2% formaldehyde to produce crosslinking of the DNA-protein. The Hi-C library construction process involves crosslink DNA, digestion, biotin labelling, proximity ligation, and DNA purification, was prepared following the protocol^34^. Finally, the Hi-C library was subjected to paired-end sequencing with 150 bp read lengths using the MGISEQ-2000 platform to capture the spatial interactions between chromosomal regions. As a result, 109.97 Gb of Hi-C read data was generated, with an average sequencing coverage of 111.71× (Table 1).

RNA sequencing (RNA-Seq) using short-read sequencing technology was widely used method for transcriptome profiling^35^. While emerging single molecule, long-read RNA-Seq technologies have enabled new approaches to study the transcriptome and its function^36^. SMRT isoform sequencing (Iso-Seq) with the PacBio platform can generate full-length cDNA sequences^37^. Read lengths achieved with these technologies (~15 kb) surpass lengths of most transcripts. In this study, for substantiating transcripts to annotate the genome structure, we performed RNA-Seq and Iso-Seq of the total RNA, respectively. Total RNA was extracted by grinding tissue in TRIzol reagent (Tiangen) on dry ice and processed following the protocol provided by the manufacturer. The integrity of the RNA was determined with the Agilent 2100 Bioanalyzer (Agilent Technologies) and agarose gel electrophoresis. The purity and concentration of the RNA were determined with the Nanodrop (Thermo Fisher Scientific) and Qubit (Thermo Fisher Scientific). Then, equal amount of them were pooled together for RNA sequencing. Finally, sequencing of RNA-Seq and Iso-Seq were performed on the MGISEQ-2000 platform and the PacBio Sequel II platform, respectively. A total of 17.79 Gb RNA-seq data and 64.59 Gb clean Iso-Seq data were generated (Table 1), which were then used for whole-genome protein-coding gene prediction.

### Genome survey

The k-mer analysis was performed using MGI paired-ended sequenced raw reads prior to genome assembly to estimate the genome size and heterozygosity. Briefly, 56.71 Gb raw dara was filtered by fastp v 0.21.0^38^ software with parameters of “-n 0 -f 5 -F 5 -t 5 -T 5 -q 20”, and 52.65 Gb clean data were retained (Table 1). The quality-filtered clean reads were subjected to k-17mers frequency distribution and heterozygosity using the KMC program^39^ with parameters of “-k17 -ci1 -cs1000000”. The genome size was estimated using FindGSE software^40^ and GenomeScope (v 1.0.0)^41^ with parameters of “default”. Finally, a total of 34,657,425,513 *k*-mers were counted with a *k*-mers peak at a depth of 35 (Table 2). We estimated that the genome size of the yellow grouper = K-mer num/K-mer depth = 990.21 Mb. The heterozygosity rate was estimated to be approximately 0.40% on *k*-mer depth distribution (Table 2).

**Table 2:** The result of k-mer analysis.

K-mer	K-mer num	K-mer depth	Genome size (bp)	Heterozygous ratio (%)
17	34,657,425,513	35	990,212,157	0.40%

### *De novo* assembly of the yellow grouper genome

The raw PacBio CCS reads data was used for *de novo* genome assembly using hifiasm v 0.19.4^42^ with default parameters. To further improve the accuracy of the assembly, the preliminary assembled genome was polished by short reads from the same individual using four iterative correction rounds of Nextpolish (v1.2.4^43^) with default parameters. To evaluate the accuracy of the assembly, all the Illumina paired end reads were mapped to the assembled genome using BWA (Burrows-Wheeler Aligner, v 0.7.12-r1039^44^) and the mapping rate as well as genome coverage of sequencing reads were assessed using Minimap2 v r41^45^ with parameters of “-x map-pb”. Besides, base accuracy of the assembly was calculated with samtools v 1.4^46^ and Bcftools v1.8.0^47^ with default parameters. To avoid including mitochondria sequences in the assembly, the draft genome assembly was submitted to the NT library and aligned sequences were eliminated using the blast v2.9^48^. The resulting assembly consists of 64 contigs and has a total length of 984.53 Mb with a contig N50 length of 40.27 Mb (Table 3).

**Table 3:** Assembly statistics of yellow grouper.

Genome Type	Before Hi-C				After Hi-C				
Statistical Type	Contig Length	Contig Number	Scaffold Length	Scaffold Number	Contig Length	Contig Number	Scaffold Length	Scaffold Number	Gap Length
N50	40270501	12	40270501	12	39766707	12	41385296	11	100
N60	39633425	14	39633425	14	39307284	14	40270501	14	100
N70	36808049	17	36808049	17	36720144	17	39766707	16	100
N80	35383561	19	35383561	19	33647458	20	37365810	19	100
N90	24585216	23	24585216	23	24585216	23	35383561	21	100
Longest	48783561	1	48783561	1	48783561	1	48783561	1	100
Total	984527537	66	984527537	66	984481122	65	984482722	49	1600
Length > = 1 kb	984527537	66	984527537	66	984481122	65	984482722	49	0
Length > = 2 kb	984527537	66	984527537	66	984481122	65	984482722	49	0
Length > = 5 kb	984527537	66	984527537	66	984481122	65	984482722	49	0

### Pseudochromosome construction

**Fig. 2:**
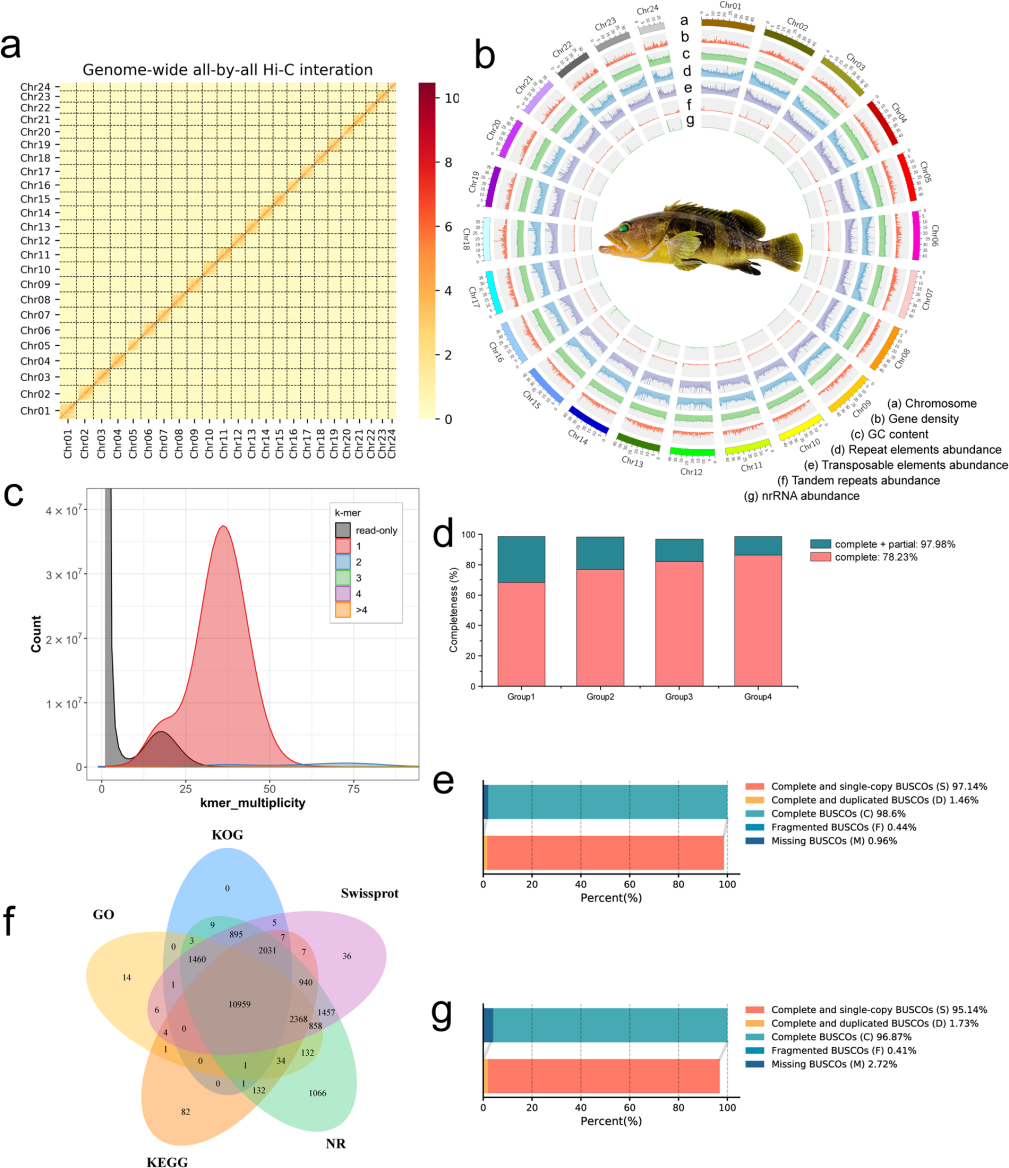
Chromosome-level genome assembly and annotation of the yellow grouper. (**a**) Hi-C interaction heat map (100-kb resolution) for the assembled genome of yellow grouper. Abscissa and ordinate represent order of each bin on corresponding chromosome group. Color block illuminates intensity of interaction from yellow (low) to red (high). (**b**) Genomic landscape of yellow grouper. All represented in 50-kb genomic windows. (**c**) The genome completeness and QV was assessed using Merqury. (**d**) CEGMA assesses the assembly status of core genes within the assembled genome. (**e**) BUSCO evaluation on the genome assembly completeness of the assembled genome. (**f**) Venn diagram of function annotations from various databases. The Venn diagram displays the overlap and uniqueness of functional gene annotations derived from five databases: SwissProt, Non-Reduntant Protein Database (NR), Kyoto Encyclopedia of Gene and Genomes (KEGG), Eukaryotic Orthologous Groups of protein (KOG) and Gene Ontology (GO). (**g**) BUSCO evaluation of gene annotation in yellow grouper.

In total, 109.97 Gb clean paired-end reads were generated from the libraries. Firstly, low-quality sequences (quality scores <20), adaptor sequences and sequences shorter than 30 bp were filtered out using fastp v0.21.0^38^ with default parameters. Then, the clean paired-end reads were mapped to the draft assembled sequence using bowtie2 v 2.3.2^49^ with parameters of “-end-to-end,–very-sensitive -L 30” to get the unique mapped paired-end reads. Valid interaction paired reads (invalid read pairs, including dangling-end, self-cycle, re-ligation, and dumped products were filtered) were identified and retained by HiC-Pro v 3.1.0^50^ from unique mapped paired-end reads for further analysis. The scaffolds were further clustered, ordered, and oriented scaffolds onto chromosomes by Lachesis^51^ with parameters of CLUSTER MIN RE SITES = 100, CLUSTER MAX LINK DENSITY = 2.5, CLUSTER NONINFORMATIVE RATIO = 1.4, ORDER MIN N RES IN TRUNK = 60, and ORDER MIN N RES IN SHREDS = 60. Finally, placement and orientation errors exhibiting obvious discrete chromatin interaction patterns were manually adjusted. Following the scaffolding procedure, the 974.86 Mb were successfully anchored to the 24 chromosomes with an integration efficiency of 99.02%, and the lengths of chromosomes ranged from 23.08 Mb to 48.78 Mb (Table 4). After Hi-C scaffolding, the 984.48 Mb chromosome-level genome of yellow grouper was assembled, with a contig N50 length of 39.77 Mb and a scaffold N50 length of 41.39 Mb (Table 3). Moreover, we evaluated the result of Hi-C based pseudo-chromosomes construction. The 24 scaffolds are clearly distinguishable in the heatmap, the interaction signal around the diagonal is strongly apparent (Fig. 2a), indicating the high-quality of the pseudochromosomes assembly.

**Table 4:** Statistics of yellow grouper genome sequence length (chromosome level).

Chromosome ID	Scaffold Length	Scaffold Number
Chr01	48,783,561	1
Chr02	48,723,295	3
Chr03	48,636,846	1
Chr04	44,928,302	1
Chr05	44,574,860	1
Chr06	44,496,831	2
Chr07	44,445,904	1
Chr08	44,294,130	2
Chr09	44,239,821	2
Chr10	42,811,158	1
Chr11	41,385,296	1
Chr12	40,500,668	1
Chr13	40,350,286	2
Chr14	40,270,501	1
Chr15	40,221,966	2
Chr16	39,766,707	1
Chr17	39,633,425	1
Chr18	39,416,018	4
Chr19	37,365,810	1
Chr20	36,720,144	1
Chr21	35,383,561	1
Chr22	32,457,409	3
Chr23	32,381,872	2
Chr24	23,076,275	4
Total	974,864,646	40

### Repeat annotation

We first annotation the tandem repeats, including simple repeat sequences (SSRs) and tandem repeat elements, were identified using the software GMATA v2.2^52^ and Tandem Repeats Finder (TRF V 4.07b^53^) with default parameters. Then, transposable elements (TE) in the yellow grouper genome were identified using a combination of *ab initio* and homology-based methods. Briefly, an *ab initio* repeat library was first predicted using MITE-hunter^54^ with parameters of “-n 20 -P 0.2 -c 3” and RepeatModeler version open-2.0.4^55^ with parameters of “-engine wublast”, in which LTR_FINDER^56^, LTRharverst^57^ and LTR_retriver^58^ synchronously to detect repeat sequences in the yellow grouper genome. The obtainted library was then aligned to TEclass Repbase (http://www.girinst.org/repbase) to classify the type of each repeat family using TEclass v 2.1.3^59^. For further identification of the repeats throughout the genome, RepeatMasker (open-4.1.4)^60^ was applied to search for known and novel TEs by mapping sequences against the *de novo* repeat library and Repbase TE library with parameters of “nolow -no_is -gff -norna -engine abblast -lib lib”. Overlapping transposable elements belonging to the same repeat class were collated and combined. A total of 405.30 Mb sequences, 41.17% of the yellow grouper genome, were identified as repeat elements (Table 5 and Fig. 2b). We estimated that the yellow grouper genome consists of approximately 0.73% tandem repeats, including 0.18% of SSR and 0.56% of tandem repeats in the genome (Table 5 and Fig. 2b). A significant portion of the genome, approximately 35.68%, is masked, resulting in 351.25 Mb being identified as transposable elements (Table 5 and Fig. 2b). Among these transposable elements, DNA transposons were the main type, occupying 20.08% (197.69 Mb) of the genome. Retroelements, including long interspersed nuclear elements (LINEs, 7.52%), long terminal repeats (LTRs, 4.24%), and short interspersed nuclear elements (SINEs, 1.02%), resulting the proportion of 12.78% of the genome (Table 5).

**Table 5:** Repetitive elements sequence statistics of the assembled genome.

Type				Number of elements	Length of sequence (bp)	Percentage of sequence (%)
TEs	ClassI: Retroelement		LINE	345,309	74,062,825	7.52
			LTR	328,360	41,779,555	4.24
			SINE	89,641	10,003,915	1.02
			Total	763,310	125,846,295	12.78
	ClassII: DNA transposon		DNA	1,526,724	197,688,186	20.08
			RC	56,005	9,574,585	0.97
			MITE	82,574	18,137,830	1.84
			Total	1,665,303	225,400,601	22.9
	Total TEs			2,428,613	351,246,896	35.68
Tandem Repeats		SSR		140,494	1,644,652	0.17
		tandem repeat elements		55,551	5,494,948	0.56
		total		196,045	7,139,600	0.73
Simple repeats				16,242	2,276,549	0.23
Other				16,406	1,844,129	0.19
Unknown				307,463	42,630,190	4.33
Low complexity				827	162,195	0.02
Total Repeats				2,965,596	405,299,559	41.17

### Gene model prediction and functional annotations

We conducted protein-coding gene prediction with three independent approaches, including homolog protein, transcriptome-assisted annotation, and *de novo* prediction, were used for gene prediction in a repeat-masked genome. For homology-based gene prediction, we utilized GeMoMa v1.6.1^61^ with default parameters to align the protein-coding sequences frome *E. fuscoguttatus* (brown-marbled grouper^9^), *E. moara* (kelp grouper^10^), *E. lanceolatus* (giant grouper^11^), *Cromileptes altivelis* (humpback grouper^18^), *Plectropomus leopardus* (leopard coral grouper^16^), *Danio rerio* (zebrafish, GCF_000002035.6^62^), and *Oryzias latipes* (Japanese medaka, GCF_002234675.1) to the genome assembly, and then got the gene structure information. For transcriptome-based prediction, the filtered long read Iso-seq and short-read RNA-seq data were aligned to the reference genome using STAR v2.7.3a^63^, followed by the transcripts were then assembled using Stringtie v1.3.4d^64^ and open reading frames (ORFs) were predicted using PASA v2.3.3^65^ to produce a training set. For the *de novo* prediction, Augustus v3.3.1^66^ with parameters of “--gff3 = on --hintsfile = hints.gff --extrinsicCfgFile = extrinsic.cfg --allow_hinted_splicesites = gcag,atac–min_intron_len = 30 --softmasking = 1” were then utilized for ab initio gene prediction with the training set. Finally, EVidenceModeler (EVM, v1.1^65^) was used to produce an integrated gene set of which gene with TE were removed using TransposonPSI package (http://transposonpsi.sourceforge.net/) and the miscoded genes were further filtered. Untranslated regions (UTRs) and alternative splicing regions were determined using PASA v2.3.3^65^ based on RNA-seq assemblies. We retained the longest transcripts for each locus, and regions outside of the ORFs were designated UTRs. Furthermore, we performed functional annotation of the predicted protein-coding genes via assigning by comparing with public databases including SwissProt^67^, the NCBI non-reduntant protein database (NR), Kyoto Encyclopedia of Gene and Genomes (KEGG)^68^, Eukaryotic Orthologous Groups of protein (KOG)^69^, and Gene Ontology (GO)^70^. The putative domains and GO terms of genes were identified using the InterProScan program with default parameters. For the other four databases, BLASTp (https://blast.ncbi.nlm.nih.gov/Blast.cgi) was used to compare the EVidenceModeler-integrated protein sequences against the four well-known public protein database with parameters of “-evalue 1e-5, -max_target_seqs. 1”^65^. Results from the five database searches were concatenated using EVidenceModeler v1.1^65^.

A total of 24,541 protein-coding genes were successfully predicted within the genome, with an average gene length and an average CDS length of 20,681.6 bp and 1,743.35 bp in each gene, respectively. The average exons number of 10.22, average exon length of 170.5 bp and average intron length of 2,052.99 in each gene (Table 6 and Fig. 2b). Further, 22,509 genes were successfully annotated, accounting for 91.72% of all predicted genes (Table 7 and Fig. 2f).

**Table 6:** Statistical results of gene structure prediction.

	Gene set	Total number of genes	Average gene length(bp)	Average CDS length(bp)	Average exons number per gene	Average exon length(bp)	Average intron length(bp)
Homoloy	*E. fuscoguttatus*	52,268	26,386.35	1,645.82	9.04	182.1	3,077.95
	*E. lanceolatus*	50,562	25,540.93	1,637.56	9.01	181.75	2,984.11
	*E. moara*	50,108	25,878.98	1,652.96	9.11	181.44	2,987.16
	*P. leopardus*	45,408	27,483.59	1,614.31	9.27	174.06	3,126.33
	*O.latipes*	51,113	34,420.92	1,707.96	9.32	183.2	3,930.43
	*D._rerio*	85,326	39,807.85	1,612.08	8.09	199.31	5,388.51
	GeMoMa	29,578	22,789.1	1,696.47	9.03	187.89	2,627.03
Transcriptome	NGS RNA seq	23,057	24,014.03	3,763.66	11.58	325.0	1,913.95
	PacBio ISO seq	37,296	18,867.52	2,714.24	9.33	290.81	1,938.39
	PASA	33,141	22,584.13	3,543.31	10.58	335.04	1,988.42
*De novo*	AUGUSTUS	25,911	20,806.43	1,714.77	10.36	165.44	2,038.71
Final	EVM	24,541	20,681.6	1,743.35	10.22	170.5	2,052.99

**Table 7:** Summary of gene annotation in the assembled genome.

Type		Number	Percent (%)
Annotation	Swissprot	21,034	85.71
	KEGG	16,567	67.51
	KOG	15,372	62.64
	GO	15,841	64.55
	NR	22,346	91.06
Total	Annotated	22,509	91.72
	Gene	24,541	100.00

To obtain the non-coding RNA (ncRNA), two strategies were used: searching against database and prediction with model. Transfer RNAs (tRNAs) were predicted using tRNAscan-SE v2.0^71^ with parameters “–thread 4 -E -I”. Micro RNA (miRNA), rRNA, small nuclear RNA, and small nucleolar RNA were detected using “cmscan” subprogram from Infernal v1.1.2^72^ to search the Rfam database^73^ with following parameter. The rRNAs and their subunits were predicted using RNAmmer v1.2^74^ with parameters “-S euk -m lsu,ssu,tsu -gff”. As a result, we annotated 1,295 rRNA, 1,946 miRNA, 276 regulatory and 2,391 tRNA (Table 8 and Fig. 2b).

**Table 8:** Statistics of annotated non-coding RNAs.

Type		Copy Number	Average Length (bp)	Total Length (bp)	Percentage of sequence (%)
rRNA (1,295)	18S	35	1,834.43	64,205	0.0065
	28S	35	4,683.06	163,907	0.0166
	5.8S	11	153.45	1,688	0.0002
	5S	1,214	114.94	139,535	0.0142
snRNA (1,946)	snRNA	224	123.72	27,713	0.0028
	miRNA	977	86.87	84,875	0.0086
	spliceosomal	659	154.98	102,131	0.0104
	other	86	168.48	14,489	0.0015
Regulatory	cis-regulatory elements	276	52.61	14,521	0.0015
tRNA	tRNA	2,391	75.77	181,161	0.0184

## Data Records

The raw sequence data, including the PacBio long-read data, MGI short-read genomic sequencing data, Hi-C data and Transcriptomic sequences, (including RNA-Seq and Iso-Seq data), have been deposited in the Genome Sequence Archive (GSA^75^) in National Genomics Data Center^76^ under the accession CRA013097^77^. Additionally, the raw data has also been deposited at NCBI with the accession number SRP479893^78^. The assembled genome sequences have been deposited in the NCBI GenBank with the accession number GCA_035609425.1^79^. The whole genome sequence data and the genome annotation files reported in this paper have been deposited in the Genome Warehouse in National Genomics Data Center^76,80^, Beijing Institute of Genomics, Chinese Academy of Sciences / China National Center for Bioinformation, under accession number GWHEQBJ00000000^81^.

## Technical Validation

Assembly completeness and accuracy were evaluated by multiple methods. First, the MGI short-read clean reads and PacBio long-read data (Table 1), were re-mapped onto the assembly using BWA v 0.7.12-r1039^44^ and minimap2^45^, respectively. The coverage rate of MGI WGS short-read and PacBio CCS long-read reached 98.87% and 97.66% of the assembly have at least 20× coverage, respectively (Table 9), demonstrating a high level of assembly accuracy. Then, the Merqury v1.3^82^ was used to assess the genome quality, with consensus quality value (QV) and completeness statistic values of 52.10 and 91.84%, respectively, indicating a high level of accuracy and completeness in the assembled genome (Fig. 2c). The CEGMA v3^83^ was employed to assess the accuracy and completeness of core genes within the assembled genome. A total of 243 core genes were assembled, accounting for 97.98% of the expected core genes. Among these, 194 were fully assembled, representing 78.23% completeness, indicating a relatively comprehensive representation of core genes in the assembled genome (Fig. 2d). Benchmarking Universal Single-Copy Orthologues (BUSCO) software v5.3.1^84^ also used to evaluate the completeness of the assembly with parameters “-l actinopterygii_odb10 -g genome”. We identified 3589 complete BUSCOs (98.60%) out of the 3640 BUSCO groups, including 3536 complete and single-copy BUSCOs (97.14%) and 53 complete and duplicated BUSCOs (1.46%). The number of fragmented BUSCOs and missing BUSCOs was 16 (0.44%) and 35 (0.96%), respectively (Fig. 2e).

**Table 9:** The alignment of short and long-read genome sequencing to the assembled genome.

Data type	Depth(X)	Base number	Coverage ratio (%)
MGI WGS short-read data	1	984,264,308	99.97
	5	983,494,266	99.90
	10	982,040,399	99.75
	20	973,423,816	98.87
PacBio CCS long-read data	1	984,522,462	100.00
	5	983,927,732	99.94
	10	982,133,510	99.76
	20	961,445,821	97.66

Furthermore, the completeness of gene annotations were evaluated using BUSCO v5.3.1^84^ with the actinopterygii_odb10 database. The annotated genes covered a total of 96.87% (3526) of the complete vertebrate core gene set, indicating a high reliable of the gene prediction results (Fig. 2g). RNA-Seq reads were mapped with the annotation results, we used Stringtie v1.3.4d^64^ with default parameters and achieved an overall mapping rate of 91.76%. Next, we compared the number of genes, gene length, coding DNA sequence (CDS) length, exons number per gene, exon length, and intron length with those of other teleost fish species (Table 10).

**Table 10:** The comparison of gene models annotated from the yellow grouper genome with those from teleost fishes.

Species	Total number of genes	Average transcript length (bp)	Average CDS length (bp)	Average exons number per gene	Average exon length (bp)	Average intron length (bp)
*E. awoara*	24,541	20,681.6	1,743.35	10.22	170.5	2,052.99
*E. fuscoguttatus*	23,748	22,913.69	1,804.35	10.37	173.97	2,252.45
*E. lanceolatus*	23,699	22,491.65	1,769.85	10.21	173.42	2,250.97
*E. moara*	23,570	22,128.05	1,790.3	10.31	173.73	2,185.64
*P. leopardus*	24,310	16,613.4	1,632.28	9.57	170.56	1,748.02
*D. rerio*	32,077	26,260.74	1,685.82	9.36	180.2	2,941.3
*O. latipes*	21,983	17,047.93	1,832.0	10.56	173.42	1,590.96

**Fig. 3:**
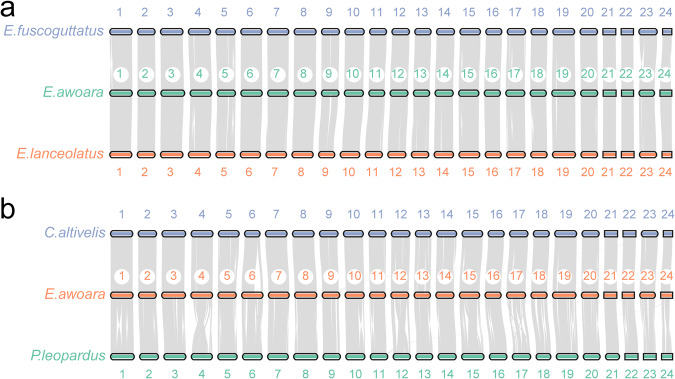
Genome collinearity between the yellow grouper and other grouper species. (**a**) Collinearity analysis within the genus. (**b**) Collinearity analysis across genera.

Genome collinearity analysis and visualizations were performed using the MCScan tool from jcvi v1.3.8^85^, obtained from https://github.com/tanghaibao/jcvi/wiki/MCscan-(Python-version). We illustrated the collinearity between the yellow grouper genome and other grouper species using collinearity plots. The yellow grouper genome demonstrates strong collinearity with related species within its genus and with the humpback grouper (*C. altivelis*) from a distinct genus (Fig. 3a,b). However, compared to another genus, the leopard coral grouper (*P. leopardus*), it exhibits more frequently chromosomes are rearranged. (Fig. 3b).

## Data Availability

No custom code was used in this study. All bioinformatics tools, commands and pipelines used in data processing were executed following the manual and protocols provided by the respective software developers. The versions of the software used, along with their corresponding parameters, have been thoroughly described in the Methods section.
